# Efficacy of the Radical Scavenger, Tempol, to Reduce Inflammation and Oxidative Stress in a Murine Model of Atopic Dermatitis

**DOI:** 10.3390/antiox12061278

**Published:** 2023-06-15

**Authors:** Alessio Ardizzone, Alberto Repici, Anna Paola Capra, Federica De Gaetano, Valentina Bova, Giovanna Casili, Michela Campolo, Emanuela Esposito

**Affiliations:** Department of Chemical, Biological, Pharmaceutical and Environmental Sciences, University of Messina, Viale Ferdinando Stagno d’Alcontres, 98166 Messina, Italy; aleardizzone@unime.it (A.A.); alberto.repici@studenti.unime.it (A.R.); annapaola.capra@unime.it (A.P.C.); federica.degaetano@unime.it (F.D.G.); valentina.bova@unime.it (V.B.); gcasili@unime.it (G.C.); eesposito@unime.it (E.E.)

**Keywords:** atopic dermatitis, tempol, inflammation, NF-kB, oxidative stress, Nrf2

## Abstract

Atopic dermatitis (AD) is the most common chronically relapsing inflammatory skin disease, predominantly common in children; it is characterized by an eczematous pattern generally referable to skin dryness and itchy papules that become excoriated and lichenified in the more advanced stages of the disease. Although the pathophysiology of AD is not completely understood, numerous studies have demonstrated the complex interaction between genetic, immunological, and environmental factors, which acts to disrupt skin barrier function. Free radicals play a key role by directly damaging skin structure, inducing inflammation and weakening of the skin barrier. Tempol (4-hydroxy-2,2,6,6-tetramethylpiperidine-1-oxyl) is a membrane-permeable radical scavenger, known to be a stable nitroxide, which exhibits excellent antioxidant effects in several human disorders, such as osteoarthritis and inflammatory bowel diseases. Considering the few existing studies on dermatological pathologies, this study aimed to evaluate tempol, in a cream formulation, in a murine model of AD. Dermatitis was induced in mice via dorsal skin application of 0.5% Oxazolone, three times a week for two weeks. After induction, mice were treated with tempol-based cream for another two weeks at three different doses of 0.5%, 1% and 2%. Our results demonstrated the ability of tempol, at the highest percentages, to counteract AD by reducing the histological damage, decreasing mast cell infiltration, and improving the skin barrier properties, by restoring the tight junction (TJs) and filaggrin. Moreover, tempol, at 1% and 2%, was able to modulate inflammation by reducing the nuclear factor kappa-light-chain-enhancer of the activated B cell (NF-κB) pathway, as well as tumor necrosis factor (TNF)-α and interleukin (IL)-1β expression. Topical treatment also attenuated oxidative stress by modulating nuclear factor erythroid 2-related factor 2 (Nrf2), manganese superoxide dismutase (MnSOD), and heme oxygenase I (HO-1) expression levels. The obtained results demonstrate the numerous advantages provided by the topical administration of a tempol-based cream formulation, in reducing inflammation and oxidative stress through modulation of the NF-κB/Nrf2 signaling pathways. Therefore, tempol could represent an alternative anti-atopic approach to treating AD, thereby improving skin barrier function.

## 1. Introduction

Atopic dermatitis (AD) is the most common chronically relapsing inflammatory skin disease, characterized by an eczematous pattern generally referred to as skin dryness and itchy papules that become excoriated and lichenified in the advanced stages of the disease [1,2]. AD generally occurs in early childhood; in fact, it has a prevalence of 10–20%. However, it can also arise in adulthood (1–3%), with an increasing worldwide incidence in recent decades [3]. Although the etiology of AD is not completely understood, numerous studies have demonstrated the complex interactions among genetic, immunological, and environmental factors that disrupt skin barrier function and elicit an epidermal inflammatory response [4]. For instance, current findings advise that the aryl hydrocarbon receptor (AHR)/AHR-nuclear translocator (ARNT) axis possesses therapeutic implications for treating inflammatory skin disease issues, providing more evidence of the critical function of filaggrin [5,6]. In fact, the activation of the AHR/ARNT axis is involved in skin homeostasis, and its pharmacological modulation enhances skin barrier functions and accelerates epidermal differentiation by increasing filaggrin expression [5,6].

Inflammation worsens the AD condition by favoring immune cell infiltration and inducing the release of several pro-inflammatory proteins such as cytokines, chemokines, and enzymes [7]. These inflammatory mediators cooperatively lead to spongiosis, hyperkeratosis, and the loss of stratum corneum [8].

Considering the key role of immunity and inflammation in the pathological context of AD, most of the drugs currently used in therapy have anti-inflammatory or immunomodulating capacities. Indeed, drugs such as corticosteroids, cyclosporine, and methotrexate represent a standard of care in the therapeutic regimen of AD patients [9]; however, their use in long-term therapies has shown many undesirable side effects [10].

To overcome this clinical challenge, scientific research has focused on understanding the pathophysiological mechanisms underlying AD, and identifying some key molecules in the inflammatory cascade triggering the pathology. As a result, new drugs directed against specific molecular targets, such as monoclonal antibodies or Janus kinase (JAK) inhibitors, were produced [11]. In this regard, clinical trials have validated the safety and efficacy of the JAK inhibitors upadacitinib and abrocitinib, which have a positive benefit–risk profile in adults and adolescents with moderate-to-severe atopic dermatitis [12,13,14]. Particularly, patients receiving upadacitinib 30 mg once daily had a quick decline in the pruritus numerical rating scale (a measure of itch intensity) and maintained a favorable clinical response for 16 weeks after starting the therapy [15].

Likewise, AD patients receiving dupilumab and tralokinumab, monoclonal antibodies that block the signaling of Th2 cytokines, also experienced a marked and quick improvement in all of the assessed measures of disease activity [16,17,18].

Nevertheless, while on the one hand, the role of inflammatory processes in the context of AD has been well established, few treatments have been directed towards oxidative and nitrosative stress.

Of interest, the most recent advances have highlighted a considerable interplay between inflammation and oxidative stress, elucidating the negative impact of oxidative damage on skin barrier impairment during cutaneous diseases and especially in AD [19].

In particular, chronic skin inflammation was associated with the overproduction of reactive oxygen species (ROS) such as superoxide and hydrogen peroxide, modifying the balance with endogenous antioxidant defenses [20]. Moreover, long-lasting oxidative conditions can affect important cellular components such as proteins, DNA, and membrane lipids, causing tissue damage and cell death through lipid peroxidation events [20].

These assumptions present a new scenario for skin disease treatments, suggesting the use of antioxidant compounds as a possible effective therapeutic strategy to improve AD symptomatology.

Here, we focused on tempol (4-hydroxy-2,2,6,6-tetramethylpiperidine-1-oxyl), a molecule classifiable as a piperidine nitroxide that has demonstrated different beneficial effects thanks to its powerful antioxidant abilities [21]. Tempol has been shown to be effective in reducing pathological features in several preclinical studies reproducing a diverse variety of human pathologies such as colitis [22], osteoarthritis [23], and myocardial ischemia [24]; however, the pharmacological activity of topically administered tempol in inflammatory skin diseases such as AD has not yet been evaluated.

Tempol’s antioxidant function was associated with superoxide dismutase (SOD)-like activity, whereby it acts as a scavenger of free radicals and oxidizes transition metals in the reduced state [25].

Moreover, despite the fact that other antioxidant species have no intracellular activity, tempol also has the ability to penetrate the cell membrane, catalyzing the disproportionation of superoxide, modulating hydrogen peroxide metabolism, and inhibiting the Fenton reaction [26].

From this perspective, a topical formulation of tempol was developed, conjugating this biologically active compound with emollient base components in order to optimally deliver the drug directly to skin lesions. Using an in vivo model of AD induced by oxazolone, a well-known skin sensitizer that mimics the dermatitis phenotype, the current study aimed to evaluate the effectiveness of this novel tempol-based cream in improving skin barrier function, while investigating the molecular mechanisms that reduce skin inflammation.

## 2. Materials and Methods

### 2.1. Materials

Tempol and all the used compounds were obtained from Sigma-Aldrich (Milan, Italy), and all the chemicals were of the highest commercial grade available. The water used throughout the study was double-distilled, then filtered through 0.22 µm Millipore^®^ GSWP filters (Bedford, MA, USA). All stock solutions were prepared in non-pyrogenic saline (0.9% NaCl; Baxter, Liverpool, UK).

### 2.2. Animals

Pathogen-free 5-week-old male SKH-1 hairless mice (Envigo, Milan, Italy) were used for this study. The animals were housed in a proper room (22 ± 2 °C, 55% ± 15% relative humidity, 12 h light/dark cycle) with water and rodent feed ad libitum. Before starting the study, the animals were maintained in a quarantine area for one week. During this stage, they were observed daily. Moreover, a numbered tag placed through the edge of the right ear identified the animals selected for the study. The animal study was performed in accordance with Italian regulations on the use of animals (D.M.116192) and Directive legislation (EU) (2010/63/EU) and ARRIVE guidelines.

### 2.3. Tempol Cream Formulation

The preparation method, according to the current European Pharmacopoeia 11th Edition (Ph. Eur.; Semi-solid preparations for cutaneous application), takes glycerine monostearate 10% (*w*/*w*), white 3aseline 25% (*w*/*w*), cetylstearyl alcohol 8% (*w*/*w*), tween 60.5% (*w*/*w*) and (just enough) purified water 100% (*w*/*w*) as raw materials. The cream was prepared using the following phases: (i) the aqueous phase, constituted by different concentrations of tempol (0.5%, 1% and 2% (*w*/*w*)), purified water, and glycerine monostearate; and (ii) the oil phase, constituted by white vaseline, cetylstearyl alcohol, and tween 60.

All the ingredients for the oil phase and for the water phase were weighed separately, before being heated up until the phases were clear. The aqueous phase was added to the oil phase under stirring, and the temperature was kept constant. After the addition, the mixture was cooled to room temperature and homogenized at high speed using an UltraTurrax T 25 (IKA-Werke, Staufen, Germany) at 11,000 rpm for 5 min.

### 2.4. Induction of AD-Like Skin Lesions

The oxazolone-induced AD model was prepared as previously reported [27] and briefly described below.

The animals were subjected to AD induction via dorsal application of 200 µL of oxazolone at 0.5% thrice weekly over a period of four weeks, while the control groups were treated with the vehicle (Tween 80) or a different percentage of tempol, three times a week for a period of four weeks (groups 1–4). Two weeks following oxazolone treatment, the onset of dermatitis was evaluated via macroscopic examination (e.g., erythema). Once confirming AD, mice were randomly divided into four experimental groups (groups 5–8) to start the treatments for the other two weeks. During these two weeks, groups 5–8, together with treatment (except for group 5, which received the vehicle), continued the treatment with 200 µL of oxazolone at 0.5% thrice weekly.

### 2.5. Experimental Groups

Mice were randomly divided into eight groups, as reported in Table 1.

Tempol doses were based on previous in vivo studies [28] and a large-scale dose study performed in our laboratory. The experimental data regarding the sham groups treated with tempol were reported only in the macroscopical evaluation and behavioral test; however, since they were comparable to the Sham group, they were omitted. Erythema and pruritogen-induced scratching behavior were measured at the end of the experiment, then the animals were sacrificed. Skin samples were surgically removed and fixed in 10% formalin for histological examinations or stored at −80 °C for molecular biology analyses. Moreover, we collected blood for further biochemical analysis.

### 2.6. Erythema Index and Pruritogen-Induced Scratching Behavior

To assess skin erythema, a score of 0 to 4 was assigned to each animal; 0 corresponded to no evidence of erythema, 1 mild erythema, 2 clear erythema, 3 moderate erythema and 4 acute erythema.

Moreover, to assess the itching, we proceeded with a scratching behavior in which mice were placed in transparent PlexiGlass chambers and allowed to acclimatize. Scratching was evaluated every 5 min for 30 min after each treatment [29]. Both macroscopic evaluations and behavioral tests were carried out by two observers in a blinded manner.

### 2.7. Hematoxylin and Eosin Staining

Hematoxylin and eosin (H&E) staining was performed as previously reported [30]. After storing the dorsal back samples for 24 h in 10% formalin, they were dehydrated with an increasing series of alcohols and finally xylol. Subsequently, the samples were embedded in paraffin. The paraffin samples were cut to 7 µm of thickness via microtome. The obtained tissue sections, having been dewaxed in xylol and rehydrated using a descending ethanol scale, were stained using H&E (Bio-Optica, Milano, Italy) and examined with an optical microscope (Nikon Eclipse Ci-L microscope, NIKON CORPORATION, Tokyo, Japan); the figures are shown at 10× magnification. Histological analyses were carried out by two observers in a blinded manner.

### 2.8. Toluidine Blue Staining

Skin sections were stained using toluidine blue to evaluate mast cells’ degranulation, as previously described [31]. Every section of 7 µm was dewaxed using a descending scale of alcohol and then stained with Toluidine blue (Bio-Optica, Milano, Italy). The mast cell count was performed on each slide using a Nikon Eclipse Ci-L microscope. Figures are shown at 20× and 40× magnifications.

### 2.9. Immunofluorescence Analysis of Filaggrin, Zonula Occludens-1(ZO-1) and Occludin

Immunofluorescence staining was performed as previously described [32]. To proceed with immunofluorescence staining, every section was probed with the following antibodies: anti-mouse Filaggrin (1:100; sc-80609 Santa Cruz Biotechnology, Santa Cruz, CA, USA), anti-rabbit ZO-1 (1:100; 61-7300 Invitrogen Waltham, MA, USA), and anti-mouse Occludin (1:100; sc-133256 Santa Cruz Biotechnology, Santa Cruz, CA, USA) at room temperature overnight. The day after, the secondary fluorescent antibodies Alexa Flour 488 goat anti-mouse and anti-rabbit IgG (A11001 and A11008; Invitrogen) were incubated for 3 h. Then, 4′,6′-diamidino-2-phenylindole (DAPI; Hoechst, Frankfurt, Germany) was added to sections for the nuclear staining. The sections were shown at 40× magnification using a Nikon Eclipse Ci-L microscope.

### 2.10. Immunohistochemical Localization of Tumor Necrosis factor (TNF)-α and Interleukin (IL)-1β

Analysis of TNF-α and IL-1β has been performed using immunohistochemistry as previously described [33]. Sections of 7 µm were incubated overnight with anti-mouse TNF-α (1:100; sc-52746, Santa Cruz Biotechnology, Santa Cruz, CA, USA) and anti-mouse IL-1β (1:100; sc-32294, Santa Cruz Biotechnology, Santa Cruz, CA, USA). After that, the sections were carefully washed with PBS and then incubated with the secondary antibody using the VECTASTAIN Universal Quick Kit, Peroxidase, R.T.U. (PK-7800; Vector Laboratories, Burlingame, CA, USA). The reaction was revealed using the water-soluble, chromogenic substrate 3,3′-Diaminobenzidine (DAB), and counter-stained with Nuclear Fast Red. For the analysis, a Nikon Eclipse Ci-L microscope was used, and figures are shown at 20× and 40×. The percentage area of immunoreactivity (determined by the number of positive pixels) was expressed as the % of total tissue area (red staining) within five random fields at a 40× magnification, and analyzed using a computerized image analysis system (Leica QWin V3, Cambridge, UK).

### 2.11. Western Blot Analysis

After the animals’ sacrifice, skin samples were homogenized to extract the cytosolic and nuclear fractions. To measure protein content, a Bio-Rad protein assay was used, with bovine serum albumin (BSA) as standard. Subsequently, the samples were heated at 95 °C for 5 min and then loaded on a 10% SDS-PAGE gel and transferred to a polyvinylidene difluoride (PVDF) membrane; after this procedure, the membranes were blocked with 5% (*w*/*v*) nonfat dried milk in buffered saline (PM) for 1 h at room temperature [34]. The following antibodies were incubated overnight and then evaluated: anti-nuclear factor kappa-light-chain-enhancer of activated B cells (NF-κB) (1:500, Santa Cruz Biotechnology, sc-8008), anti-nuclear factor of kappa light polypeptide gene enhancer in B-cell inhibitor, alpha (IĸB-α) (1:500; Santa Cruz Biotechnology, sc-1643), anti-nuclear factor erythroid 2-related factor 2 (Nrf2) (1:500, Santa Cruz Biotechnology, sc-365949), anti-manganese superoxide dismutase (MnSOD) (1:500, Merck MilliPore), anti-heme oxygenase (HO-1) (1:500, Santa Cruz Biotechnology, sc-136960), anti-induced nitric oxide synthase (iNOS) (1:500, BD Transduction Laboratories, 0332776); anti-cyclooxygenase-2 (COX-2) (1:500, Santa Cruz Biotechnology, sc-376861) in 1× phosphate-buffered saline (PBS), 5% (*w*/*v*), non-fat dried milk, and 0.1% Tween-20. Subsequently, the membranes were washed and incubated with a secondary antibody, anti-mouse or anti-rabbit (1:1000, Jackson ImmunoResearch, West Grove, PA, USA) for 1 h at room temperature. To confirm that the samples contained the same concentration of proteins, we incubated membranes with primary antibody anti-β-actin (1:500; sc-47778; Santa Cruz Biotechnology, Dallas, TX, USA), for the cytosolic fraction, or LAMIN A/C (1:500; sc-376248; Santa Cruz Biotechnology, Dallas, TX, USA), for the nuclear fraction.

### 2.12. Myeloperoxidase (MPO) and Malondialdehyde (MDA) Assays

MPO levels were assessed as previously described [35]. The change in absorbance was calculated via a spectrophotometer at 650 nm. MPO activity was measured as the quantity of enzyme degrading 1 mM of peroxide min^−1^ at 37 °C, expressed in units per gram of wet tissue.

The level of MDA in skin samples was evaluated as evidence of lipidic peroxidation, and determined as previously described [36].

### 2.13. Nitrate and Nitrite Assay

Nitrate/nitrite levels were assessed using the Griess reaction assay kit [29] and expressed as mmol/mouse.

### 2.14. ELISA Kits

Levels of immunoglobulin E (IgE), IL-4 and IL-13 were evaluated on serum using the following ELISA kits: IgE (ab157718), IL-4 (BMS613) and IL-13 (LS-F23522). The ELISA kits were used as previously described [37] and according to the manufacturer’s protocols.

### 2.15. Statistical Analysis

The values are expressed as the mean ± standard deviation (SD) of *N* observations; each *N* represents the number of animals studied. All the results were analyzed using a one-way ANOVA followed by a Bonferroni post hoc test for multiple comparisons; only a *p*-value of less than 0.05 was considered significant.

## 3. Results

### 3.1. Effect of Cream-Based Tempol Treatment on Skin Damage following Oxazolone-Induced AD

**Figure 1:**
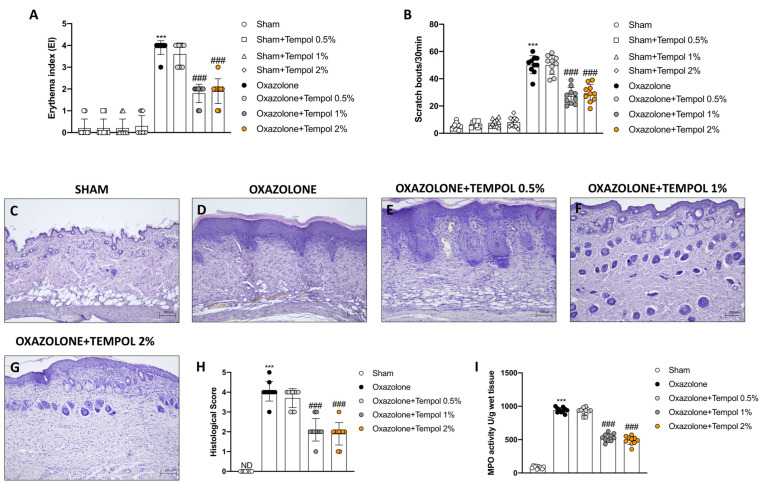
**Effect of Tempol on AD signs and histological damage induced by oxazolone**. Among three different concentrations of tempol, only tempol 1% and 2% showed significant effects, reducing both erythema and scratch bouts (**A**,**B**). While extensive damage was observed in the skin of the oxazolone group (**D**, score **H**) compared to skin tissues from sham mice (**C**, score **H**). Skin injury caused by oxazolone was notably decreased by tempol 1% (**F**, score **H**) and tempol 2% (**G**, score **H**). In addition, MPO levels were reduced in mice treated with tempol 1% and 2% (I). Differently, tempol 0.5% administration proved to be ineffective in reducing AD symptoms (**A**,**B**), histological damage (**E**, score **H**), and MPO activity (**I**). In every experimental group, the number of mice was *n* = 10. The results of histological evaluations are displayed at 10× magnification. Values are means ± SD. one-way ANOVA test. *** *p* < 0.001 vs. sham; ### *p* < 0.001 vs. oxazolone; ND: not detectable.

Macroscopic and itch evaluations showed a considerable increase in the erythema index as well as the total number of scratching bouts in oxazolone vehicle animals compared to the Sham group (Figure 1A,B). Tempol cream administration, especially at the two higher concentrations of 1% and 2%, significantly reduced the formation of erythema and the total number of scratches (Figure 1A,B).

To examine the efficacy of tempol cream on histopathological features, skin tissues of mice were stained with H&E. In the oxazolone group, histological examination of the skin revealed a marked hyperkeratosis with crusting, epidermal thickening and a considerable neutrophilic infiltration (Figure 1D, histological score H) compared to the control mice (Figure 1C, histological score H). However, topical treatment with tempol 1% and 2% restored the morphological architecture of the skin, decreasing the epidermal thickening and the presence of polymorphonuclear cells (Figure 1F,G, respectively, histological score 1H). The tempol 0.5% topical administration was ineffective in reducing skin tissue injury (Figure 1E, histological score H). The MPO assay, a reliable test to verify the index of neutrophilic infiltration, confirmed the ability of both tempol cream 1% and 2% to contain inflammatory cell infiltration in the skin compared to oxazolone-treated mice (Figure 1I). No significant results were observed following tempol 0.5% topical treatment, compared to the oxazolone-vehicle group (Figure 1I).

### 3.2. Effect of Cream-Based Tempol Treatment on Mast Cell Infiltration Following Oxazolone-Induced AD

**Figure 2:**
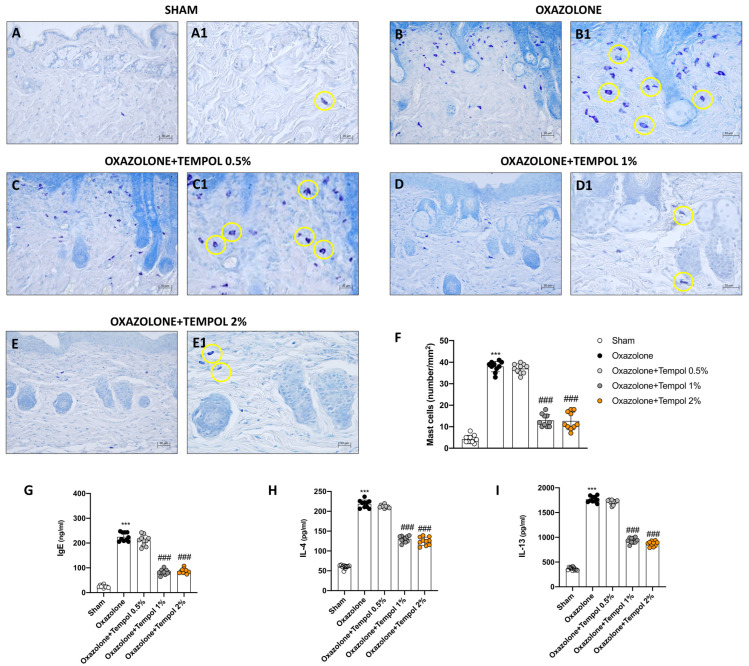
**Effect of tempol on mast cells in skin tissues after oxazolone-induced atopic dermatitis.** A significant number of mast cells were observed in oxazolone mice tissues (**B**,**B1**, score **F**) compared to the sham group (**A**,**A1**, score **F**). Tempol 0.5% treatment did not reduce mast cell infiltration (**C**,**C1**, score **F**). A marked improvement in mast cell number was observed following tempol 1% (**D**,**D1**, score **F**) and tempol 2% (**E**,**E1**, score **F**). Yellow circles indicate the mast cells. Additionally, treatment with tempol 0.5% produced no significant results on serum levels of IgE, IL-4 and IL-13 (**G**–**I**). The two concentrations of 1% and 2%, however, notably lessened serum levels of IgE, IL-4 and IL-13 (**G**–**I**). In every experimental group, the number of mice was *n* = 10. The figures are shown at 20× and 40× magnification. Values are means ± SD. One-way ANOVA test. *** *p* < 0.001 vs. sham; ### *p* < 0.001 vs. oxazolone.

**Figure 3:**
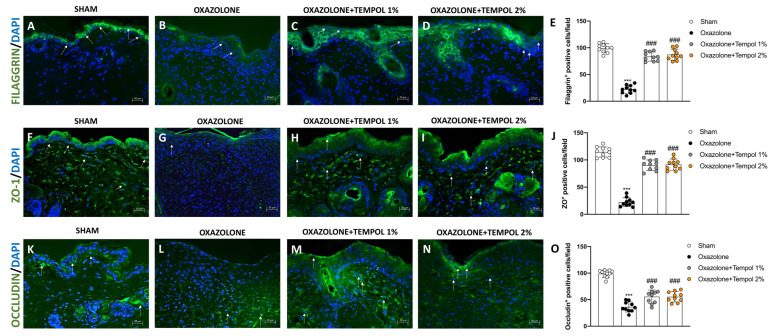
**Effect of tempol administration on filaggrin, ZO-1 and occludin expression.** Skin tissues from oxazolone-induced mice showed a lower expression of filaggrin (**B**, score **E**), ZO-1 (**G**, score **J**) and occludin (**L**, score **O**), compared to the respective sham groups (**A**, score **E**,**F**, score **J**,**K**, score **O**). Topical treatments with tempol 1% (**C**, score **E**,**H**, score **J**,**M**, score **O**) and tempol 2% (**D**, score **E**,**I**, score **J**,**N**, score **O**) notably increased the expression of filaggrin and TJs. White arrows indicate positive cells. In every experimental group, the number of mice was *n* = 10. The figures are shown at 40× magnification. Values are means ± SD. One-way ANOVA test. *** *p* < 0.001 vs. sham; ### *p* < 0.001 vs. oxazolone.

We performed toluidine blue staining to provide evidence of the presence of mast cells in skin tissues following oxazolone-induced AD. A notable increase in the number of mast cells was noticed in oxazolone-treated mice (Figure 2B,B1, score F) compared to the sham group (Figure 2A,A1, score F). Tempol topical administration at the two highest concentrations of 1% (Figure 2D,D1, score F) and 2% (Figure 2E,E1, score F) was effective in moderating mast cell infiltration. Differently, tempol 0.5% topical administration was ineffective in reducing mast cell degranulation (Figure 2C,C1, score F). We also conducted ELISA assays to assess serum levels of IgE, IL-4 and IL-13. The levels of these three factors were significantly increased in the oxazolone-treated group compared to the control animals (Figure 3G–I). Topical treatment with tempol 0.5% did not produce a statistically significant reduction in all three parameters (Figure 3G–I). However, the application of tempol 1% and 2% creams led to a considerable decrease in IgE, IL-4 and IL-13 levels in the sera of treated mice (Figure 3G–I).

Considering these preliminary results, which highlighted the inefficacy of tempol 0.5% in counteracting oxazolone-induced AD, we decided to continue our experiments studying only tempol 1% and 2%.

### 3.3. Tempol Topical Treatment Restored Skin Barrier Following Oxazolone-Induced AD

We investigated filaggrin and tight junction (TJs) expression via immunofluorescence staining in order to assess tempol’s protective properties on the skin barrier. Skin samples from sham animals showed physiological expression of filaggrin (Figure 3A, score E), ZO-1 (Figure 3F, score J), and occludin (Figure 3K, score O). Differently, oxazolone-treated mice revealed a substantial skin barrier impairment, resulting in a significant decrease in filaggrin (Figure 3B, score E) and TJs immunopositivity (Figure 3G, score J and L, score O, respectively). Tempol topical administrations at both concentrations markedly restored skin barrier function by increasing positive staining for filaggrin (Figure 3C,D, score E), as well as ZO-1 (Figure 3H,I, score J) and occludin (Figure 3M,N, score O), almost comparable to the sham group.

### 3.4. Topical Tempol Treatment Modulated the NF-κB Pathway and Proinflammatory Cytokine Overexpression Following Oxazolone-Induced AD

**Figure 4:**
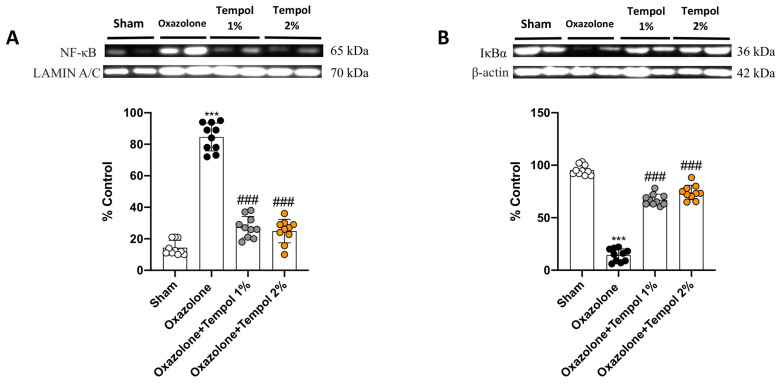
**Effect of tempol 1% and 2% treatments on NF-κB/IkB**-**α pathway.** Physiological levels of NF-κB and IkB-α were found in sham mice (**A**,**B**). Oxazolone-induced AD notably promoted the translocation of NF-κB and the degradation of IkB-α (**A**,**B**). Treatments with tempol 1% and 2% considerably reduced NF-κB expression and upregulated IkB-α levels (**A**,**B**). In every experimental group, the number of mice was *n* = 10. Values are means ± SD. One-way ANOVA test. *** *p* < 0.001 vs. sham; ### *p* < 0.001 vs. oxazolone.

The NF-κB transcription factor family plays a central role in the progression and maintenance of inflammatory status during the course of AD [38]; therefore, we assessed the potential anti-inflammatory skills of tempol cream by evaluating its action on this signaling pathway. The oxazolone challenge induced skin inflammation, leading to a notable increase in NF-κB translocation into the nucleus (Figure 4A, densitometric analysis A1) and an increased cytosolic degradation of IκB-α (Figure 4B, densitometric analysis B1), compared to the sham animals. Cream-based tempol at concentrations of 1% and 2% was able to reduce NF-κB signaling pathway activation, as shown by the Western blot analysis (Figure 4A, densitometric analysis A1 and B, densitometric analysis B1).

**Figure 5:**
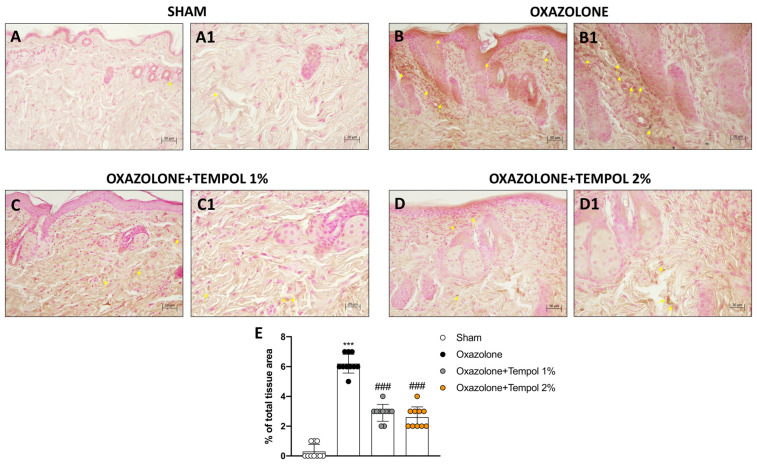
**Effects of tempol 1% and 2% administrations on TNF-α expression.** Tissue from oxazolone-induced AD mice exhibited high expression of TNF-α (**B**,**B1**, score **E**) compared to the sham animals (**A**,**A1**, score **E**). Treatments with tempol 1% (**C**,**C1**, score **E**) and tempol 2% (**D**,**D1**, score **E**) remarkably reduced TNF-α expression. Yellow arrows indicate immunopositivity. In every experimental group, the number of mice was *n* = 10. The figures are shown at 20× and 40× magnification Values are means ± SD. One-way ANOVA test. *** *p* < 0.001 vs. sham; ### *p* < 0.001 vs. oxazolone.

**Figure 6:**
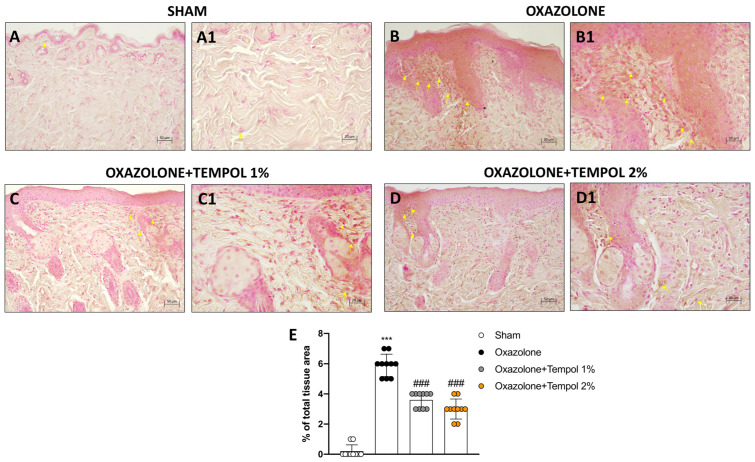
**Effects of tempol 1% and 2% administrations on IL-1β levels.** Skin samples from oxazolone-induced AD mice revealed high IL-1β positivity (**B**,**B1**, score **E**) compared to the sham animals (**A**,**A1**, score **E**). Topical treatment with tempol 1% (**C**,**C1**, score **E**) and tempol 2% considerably reduced the IL-1β expression (**D**,**D1**, score **E**). Yellow arrows indicate immunopositivity. In every experimental group, the number of mice was *n* = 10. The figures are shown at 20× and 40× magnification. Values are means ± SD. One-way ANOVA test. *** *p* < 0.001 vs. sham; ### *p* < 0.001 vs. oxazolone.

NF-κB translocation exacerbates the skin’s inflammatory processes by enhancing production of pro-inflammatory mediators such as cytokines [38]. Therefore, we analyzed the effect of tempol administration on TNF-α and IL-1β expression via immunohistochemistry. After AD induction, a substantial increase in TNF-α and IL-1β immunopositivity was detected in oxazolone-treated animals (Figure 5B,B1, score E and Figure 6B,B1, score E) compared to control mice (Figure 5A,A1, score E and Figure 6A,A1, score E). Contrarily, TNF-α and IL-1β expression were significantly reduced following tempol treatment both at 1% (Figure 5C,C1, score E and Figure 6C,C1, score E) and 2% (Figure 5D,D1, score E and Figure 6D,D1, score E).

### 3.5. Topical Tempol Treatment Attenuated AD-Induced Oxidative Stress by Modulating the Nrf2 Pathway

**Figure 7:**
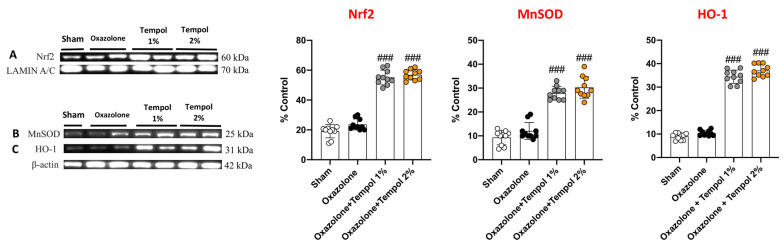
**Tempol treatments enhanced antioxidant defense.** Topical treatment with tempol at both dosages considerably increased the expressions of different factors involved in the antioxidant activity. Indeed, if compared to oxazolone mice, tempol administration led to a significant increase in the expression of the nuclear factor Nrf2 (**A**), alongside upregulating MnSOD (**B**) and HO-1 (**C**). In every experimental group, the number of mice was *n* = 10. Values are means ± SD. One-way ANOVA test. ### *p* < 0.001 vs. oxazolone.

Different pieces of evidence indicate that oxidative stress is a promotor of skin inflammation in AD [39]. In particular, it was demonstrated that Nrf2-mediated defense responses weaken skin inflammation; therefore, its activation appears to be a promising target for various skin diseases including AD [40]. Thus, to assess tempol cream’s protective action against oxidative stress, we focused on the Nrf2 pathway by employing the Western blot technique. Our data revealed that tempol 1% and 2% topical administrations enhanced antioxidant defenses by upregulating Nrf2 expression (Figure 7B and densitometric analysis B1) in the nucleus, and consequently increasing the levels of HO-1 (Figure 7C and densitometric analysis C1) and Mn-SOD (Figure 7A and densitometric analysis A1) in the cytosol. Taken as a whole, our results demonstrated the positive modulation of the Nrf2 pathway by cream-based tempol, slowing the progression of the oxazolone-induced skin damage.

### 3.6. Cream-Based Tempol Modulated the Interplay between Nitrosative Stress and Lipid Peroxidation Driven by Oxazolone-Induced AD

**Figure 8:**
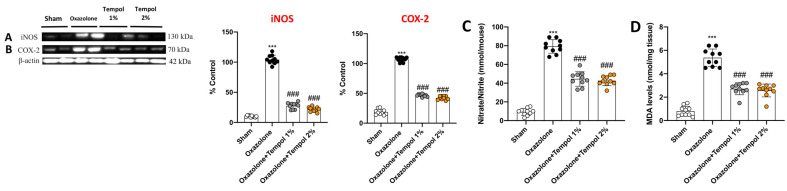
**Activity of tempol on inflammatory and oxidative stress markers.** Basal levels of iNOS and COX-2 were detected in the mice control groups; both enzymes were increased following oxazolone-induced AD (**A**,**B**). Tempol at both dosages reduced the expression of these markers (**A**,**B**). Furthermore, topical tempol treatment reduced the levels of nitrite/nitrate (**C**) and MDA (**D**), compared to oxazolone mice. In every experimental group, the number of mice was *n* = 10. Values are means ± SD. One-way ANOVA test. *** *p* < 0.001 vs. sham; ### *p* < 0.001 vs. oxazolone.

Reactive nitrogen species (RNS), lipid peroxides, and reactive metabolites are continually generated in response to environmental and endogenous pro-oxidant agents in the keratinocytes, thus worsening the pathological condition of AD [41]. Our data highlighted a noteworthy interplay between inflammation and nitrosative stress in eliciting severe skin injury, as demonstrated by the overexpression of iNOS and COX-2 in oxazolone mice compared to control animals (Figure 8A,B, respectively, see densitometric analysis A1,B1). Moreover, the severity of nitrosative stress and lipid peroxides induced by oxazolone challenge was also confirmed by the high concentrations of nitrite and MDA when compared to the sham group (Figure 8C,D). Tempol cream administrations both at 1% and 2% demonstrated good management of inflammatory and oxidative stress parameters, reducing the expression of pro-inflammatory enzymes as well as nitric species and lipidic peroxidation (Figure 8C,D).

## 4. Discussion

Tempol is a well-known molecule that acts as a free radical scavenger and nitric oxide spin trap, resulting in the effective treatment of many human disorders [42]. In particular, clinical investigations have emphasized how topical tempol treatments can be used to counter dermatoxicity and alopecia in patients after chemotherapy and radiation therapy, indicating good safety and pointing to the potential of this molecule to be a successful treatment for other skin conditions [43,44].

Based on this, we developed a cream product containing tempol and excipients, with moisturizing and emollient properties, with the purpose of testing its effectiveness in an animal model of AD caused by oxazolone and examining the signaling pathway involved in anti-inflammatory and antioxidant responses.

Erythema and pruritus are hallmarks of the skin inflammatory reactions that occur during AD; both lead to the impairment of the epidermal homeostasis, favoring the establishment of an itch–scratch cycle and resulting in cutaneous-type hypersensitivity [45]. In the present study, macroscopical evaluations and behavioral testing demonstrated that tempol was able to reduce the severe erythema and scratching caused by oxazolone challenge, thereby providing effective symptom relief.

Skin lesions typically appear in the chronic phase of AD, because of the prolonged inflammatory status which in the long-term causes hyperkeratinization, swelling, and epidermal thickness [46]. In this study, topical administration of oxazolone successfully induced AD-like skin lesions, as indicated by the presence of excoriation, edema, and dryness in epidermal tissues. By reducing the usual histopathological hallmarks of AD, such as epidermal hyperplasia and dermal edema, topical treatment with tempol substantially decreased the severity of the AD-like skin lesions. In addition, tempol administration was able to decrease neutrophils’ infiltration, as indicated by the lower levels of MPO.

In skin allergy illnesses such as AD, mast cells are recognized as important immune cells that mediate immunoglobulin E (IgE) reactions and inflammatory responses [47].

In particular, mast cells are activated by the cross-linking of allergen-bound IgE to high-affinity IgE receptors, triggering these immune cells to degranulate and release histamine, prostaglandin D2 (PGD2), and TNF-α, all of which stimulate inflammatory responses by attracting in T-helper (Th)-2 cells and secreting Th-2 cytokines [48,49].

Accordingly, our data showed a significant increase in the numbers of dermal mast cells following oxazolone-induced AD, while tempol topical administration lessened the presence of degranulating mast cells, highlighting a considerable decrease in allergic immune response which was also evidenced by the diminished levels of IgE, IL-4 and IL-13.

Th1/Th2 cell dysregulation, IgE production, and mast cell hyperactivity (occurring in AD) all negatively affect skin barrier function, which is crucial for regulating paracellular diffusion of water, solutes, and pathogens [50].

Specifically, it has been observed that loss-of-function mutations in the filaggrin gene, which can cause cellular abnormalities in keratinocytes and epidermal impairment, may be the cause of skin barrier abnormalities [51].

Moreover, in clinical specimens of AD, dysregulation of TJs, the most apical component of intracellular epithelial junctions, has been observed [52]. TJs depletion facilitates the entry of exogenous molecules into the skin, raising the risk of recurrent dermal infections. Thus, it is clear that these proteins are crucial for maintaining the integrity of the skin barrier and regulating paracellular permeability. [53].

The results obtained from this study clearly confirmed a reduction in both filaggrin and TJs expressions induced by oxazolone challenges. However, topical tempol administration acted as a barrier-restoring therapy, effectively promoting an increase in filaggrin in the cornified layer and TJs proteins in dermal tissue.

NF-κB activation is one of the critical pathways that occurs in AD; it acts by eliciting an inflammatory response that exacerbates skin damage [54]. A wide range of inflammatory cytokines and chemokines are produced as a result of NF-κB activation, which appears to be specifically associated with microbiological infection and inadequate immune response during AD [54]. Overall, these assumptions indicate that the selective inhibition of NF-κB activity could represent a valid therapeutic strategy in the management of AD.

In this study, we demonstrated a notable increase in NF-κB activity in oxazolone skin-lesioned mice, confirming the role of this transcription factor in the exacerbation of AD. Nevertheless, topical tempol treatment showed powerful anti-inflammatory properties by modulating NF-κB activation, while suppressing inflammatory cytokines such as TNF-α and IL-1β.

It has been recognized that oxidative stress promotes skin inflammation by upregulating pro-inflammatory genes and favoring injurious mechanisms that largely contribute to the pathogenesis of cutaneous disorders [39]. In this regard, recent reports have proved that the redox-sensitive transcription factor Nrf2 is involved in the regulation of epidermal homeostasis, inhibiting the inflammation and oxidative stress induced by pathological skin conditions such as AD [55]. Thus, we hypothesized that tempol might influence the signaling activity of the Nrf2 pathway, exhibiting protective benefits against skin tissue inflammation. From the obtained data, it can be deduced that tempol-based formulations promoted antioxidant defense by upregulating Nrf2 levels and cytosolic enzymes such as HO-1 and Mn-SOD, thus strengthening the positive promotion of this pathway.

Literature data provided a valuable overview regarding NF-κB/Nrf2 signaling targeting, advancing its modulation as an advantageous strategy to counteract inflammatory disorders alongside controlling nitrosative stress and lipid peroxidation [36].

In line with this, we discovered that the Nrf2 induction exerted by tempol inhibited the rise of oxidative and nitrosative damages. This was proven by the decreased levels of the pro-inflammatory enzymes iNOS and COX-2, as well as oxidative/nitrosative indicators such as nitrate and MDA, after topical administration of tempol cream in mice.

Comprehensively, our results support that tempol treatment modulates the NF-κB/Nrf2 axis to alleviate the features of AD, as well as inflammation and oxidative/nitrosative stress.

These outcomes are consistent with the latest scientific evidence in the field of dermatological research, which shows that sulforaphane and ursolic acid act as antioxidant and detoxifying agents, thereby reducing oxidative stress and inflammation by activating the Nrf2 pathway [56,57]. In light of this, we maintain that future AD-related research should consider the modulation of this biological axis, leading to the validation of further compounds, whether of natural or synthetic origin, which may have a therapeutic effect on AD-like lesions.

## 5. Conclusions

To summarize, the results obtained in the present study highlight the numerous advantages provided by the topical administration of this novel tempol-based cream formulation, proposing an alternative therapeutic approach to protecting the epidermis in the setting of pathological AD. In particular, we demonstrated tempol’s biological abilities in counteracting the progression of oxazolone-induced AD by improving skin barrier function. These beneficial outcomes resulted in a reduction in the inflammatory state and an effective enhancement of antioxidant defenses through the modulation of the NF-κB/Nrf2 signaling pathways. Therefore, this novel tempol formulation could have clinical relevance for treating AD, representing a promising approach to slowing atopic features and symptoms, thus improving patients’ quality of life.

## 6. Limitations

Despite the encouraging results of the present study, several limitations should be addressed. Firstly, even if the oxazolone-induced mouse model shows the highest degree of overlap with human AD, preclinical models are not always able to completely reproduce human illnesses, thereby limiting the translatability of the results. Indeed, new state-of-the-art methods using transcriptome analysis have deepened our comprehension of AD models by comparing the intricate molecular networks between mouse models and genes from human AD skin. The highest overlap of genes between the two species was around 40% in any model of induced AD, confirming that animal models reflect only limited aspects of human AD.

Furthermore, even though we have shown that tempol-based cream can influence the Nrf2 signaling pathway, further research employing knockout in in vivo models will be able to establish the pharmacological action of this compound more precisely in the setting of AD.

## Figures and Tables

**Table 1:** Experimental groups and procedure of the study.

Experimental Groups		Treatment
Group 1	Sham + vehicle	Mice received Tween 80 without oxazolone challenging three times a week for four weeks (*N* = 10)
Group 2	Sham + tempol 0.5%	Mice received vehicle for two weeks, then tempol 0,5% cream three times a week for the other two weeks (*N* = 10)
Group 3	Sham + tempol 1%	Mice received vehicle for two weeks, then tempol 1% cream three times a week for the other two weeks (*N* = 10)
Group 4	Sham + tempol 2%	Mice received vehicle for two weeks, then tempol 2% cream three times a week for the other two weeks (*N* = 10)
Group 5	Oxazolone + vehicle	Mice received oxazolone 0.5% three times a week for four weeks (*N* = 10)
Group 6	Oxazolone + tempol 0.5%	Mice received only oxazolone 0.5% three times a week for two weeks, then, one hour after oxazolone challenging, tempol 0.5% cream was applied on the dorsal back area for the other two weeks (*N* = 10)
Group 7	Oxazolone + tempol 1%	Mice received only oxazolone 0.5% three times a week for two weeks, then, one hour after oxazolone challenging, tempol 1% cream was applied on the dorsal back area for the other two weeks (*N* = 10)
Group 8	Oxazolone + tempol 2%	Mice received only oxazolone 0.5% three times a week for two weeks, then, one hour after oxazolone challenging, tempol 2% cream was applied on the dorsal back area for the other two weeks (*N* = 10)

## Data Availability

All the results were included in this study and available to the corresponding author’s address.
